# Applicability and prognostic value of frailty assessment tools among hospitalized patients with advanced chronic liver disease

**DOI:** 10.3325/cmj.2021.62.8

**Published:** 2021-02

**Authors:** Lubomir Skladany, Zuzana Drotarova, Janka Vnencakova, Daniela Jancekova, Pavol Molcan, Tomas Koller

**Affiliations:** 1 Division of Hepatology, Gastroenterology, and Liver Transplantation (HEGITO), Department Internal Medicine II, Slovak Medical University, F. D. Roosevelt University Hospital, Banska Bystrica, Slovakia; 2 Subdivision of Gastroenterology and Hepatology, Fifth Department of Internal Medicine, Comenius University Faculty of Medicine, University Hospital Bratislava Ruzinov, Bratislava, Slovakia

## Abstract

**Aim:**

To assess and compare the feasibility and prognostic value of various frailty assessment tools among decompensated cirrhosis inpatients.

**Methods:**

Our prospective observational registry included consecutive patients admitted for cirrhosis between June 2017 and July 2018. Exclusion criteria were intensive-care unit admission, hepatocellular carcinoma outside of the Milan criteria, and other malignancies. Frailty at baseline was assessed with the Liver Frailty Index (LFI), Clinical Frailty Scale (CFS), Fried Frailty Score (FFS), and Short Physical Performance Battery test (SPPB). The follow-up lasted for at least 180 days.

**Results:**

The study enrolled 168 patients (35.1% women, median age 57.9 years). The most frequent primary etiology was alcohol-related liver disease (78.6%). The Median Model for End-Stage Liver Disease (MELD) was 16. The 80th percentile of frailty scores was LFI>5.4, CFS>4, FFS>3, and SPPB<5, and it identified patients with higher mortality. LFI and CFS had the highest numerical prognostic value for in-hospital, and 90- and 180-day mortality. In a bivariate analysis of the risk of death or liver transplantation, the combination of MELD and LFI had the highest concordance (0.771 ± 0.04). In a multivariate model, MELD score (HR 1.17, 95% CI 1.12-1.22), overt encephalopathy (2.39, 1.27-4.48), infection at baseline (2.32, 1.23-4.34), and numerical LFI (1.41, 1.02-1.95) were independent predictors of overall mortality.

**Conclusion:**

Frailty assessment using the evaluated tools is feasible among hospitalized cirrhotic patients, identifying those with worse prognosis. CFS had the highest applicability and accuracy for the initial assessment and LFI for the initial and follow-up assessments.

Advanced chronic liver disease (ACLD) is one of the most frequent causes of premature death in Central European countries (1). Sarcopenia, as one of its most common complications, affects 40% to 70% of patients (2,3). Sarcopenia in ACLD is further complicated by the loss of physiological reserve and increased vulnerability to adverse health outcomes. The concept of physical frailty has recently been translated to hepatology from the field of geriatric medicine. Frailty is a risk factor for a further health-related decline and adverse outcomes (4). Over the last decade, it has emerged as an important force shaping the field of ACLD care. Several studies have reported that frailty increased the risk of hospitalization (5), waiting list drop-out (6), and mortality (7,8), and decreased the likelihood of liver transplantation (LT) (9). Frailty assessment tools in patients with ACLD have been recently reviewed by the American Society of Transplantation Liver and Intestinal Community of Practice (10). According to the report, it is not clear how these tools perform among inpatients. Hospitalized patients often present with a transient worsening of their physical performance, thus frailty status on admission might underestimate their true physiological reserve. In addition, several measures of physiological function have limited applicability for bed-bound patients. Hospitalization for an ACLD complication, however, represents an opportunity for early intervention. Management decisions in ACLD largely depend on the estimated prognosis and the suitability for LT. Our study therefore aimed to evaluate the feasibility and added prognostic value of frailty in hospitalized ACLD patients using several approved frailty assessment tools.

## Patients and methods

Our prospective registry for hospitalized cirrhotic patients, HEGITO7, included consecutive patients admitted for cirrhosis between June 2017 and July 2018 to the Department of Hepatology, Gastroenterology, and Transplantation (HEGITO) at the tertiary medical center with an LT program. The main inclusion criterion was an established diagnosis of cirrhosis with a decompensating event requiring hospitalization, such as ascites, portal hypertension bleeding, hepatic encephalopathy (HE), alcoholic hepatitis complicating cirrhosis, or infection. Patients with hepatocellular carcinoma (HCC) or LT candidates were included only in the presence of a decompensating event requiring hospitalization and HCC disease stage within the Milan criteria. We excluded patients hospitalized for elective treatment, those with other known malignancies, those with HCC outside the Milan criteria, patients admitted to the intensive care unit, those unable to move for reasons not related to cirrhosis, and patients who withdrew the informed consent. On admission, we recorded the following demographic and clinical variables: age, sex, etiology of cirrhosis, Model for End-Stage Liver Disease-Sodium (MELD-Na) score (calculated at http://lillemodel.com, further referred to as MELD), Child-Pugh-Turcotte score (*lillemodel.com*), C-reactive protein (CRP), grades of ascites, and grades of HE as diagnosed by clinical judgment and graded according to the West-Haven classification for HE. The number connection test was also performed. The presence of infection, such as urinary tract infection, spontaneous bacterial peritonitis, pneumonia, sepsis, soft tissue infection, etc, on admission was diagnosed according to the standard diagnostic criteria (11).

On admission, study nurses performed frailty assessment using four available diagnostic tools: Liver Frailty index (LFI), Clinical Frailty Scale (CFS), Fried Frailty Score (FFS), and Short Physical Performance Battery test (SPPB). LFI and SPPB consist of the following tests of physiological function: 1. handgrip strength (HGS) test, in which the average strength of the dominant hand from three trials, measured using a dynamometer (KERN MAP 80K1) in kilograms, is calculated; 2. chair-stands test, used to determine the time it takes for the patient to do five chair stands without the help of hands; 3. balance test, used to determine the time it takes for the patient to maintain balance having feet together, at the semi tandem, and at tandem position, for a maximum of 10 seconds; 4. gait speed test, used to measure the time it takes for the patient to walk four meters (m/s). Patients unable to stand are assigned a balance score of 0, a chair-stand score of 32 seconds, and a gait-speed score of 0. From the measured parameters, we calculated LFI using the online calculator at *liverfrailtyindex.ucsf.edu*. CFS, FFS, and SPPB test were carried out in all patients including those unable to stand-up or walk (6).

During the follow-up, we recorded the length of hospital stay (in days) and in-hospital mortality. After discharge, patients were scheduled for regular follow-up visits. Survival status was verified by comparing against the national registry of deceased inhabitants. The follow-up length was determined from the study inclusion to the day of death or LT, whichever occurred first. LT was coded depending on the type of analysis, either as LT for the overall survival in the competing events analysis, or as death in the transplant-free survival analysis (the Cox model). All other patients were censored at least after 180 days, with a median follow-up of censored patients of 300 days.

All procedures involving human participants were approved by the Ethics Committee of the F. D. Roosevelt University Hospital, and were in accordance with the 1964 Helsinki Declaration and its later amendments (www.wma.net) or comparable ethical standards. The reported clinical and research activities are consistent with the Principles of the Declaration of Istanbul, as outlined in the Declaration of Istanbul on Organ Trafficking and Transplant Tourism (12). All patients signed an informed consent before study enrolment.

### Statistical analysis

The data are presented as medians and interquartile ranges (IQR) or numbers and percentages. The cut-off values for diagnosing frailty were defined according to the 20th or 80th score percentiles and according to the cut-offs for mortality prediction identified using the area under the receiver operating curve (AUROC) analysis at various time points (in-hospital, 30, 90, 180 days) (13). From the AUROC analysis, we excluded 7 patients who underwent LT within 180 days of follow-up.

**Figure 1:**
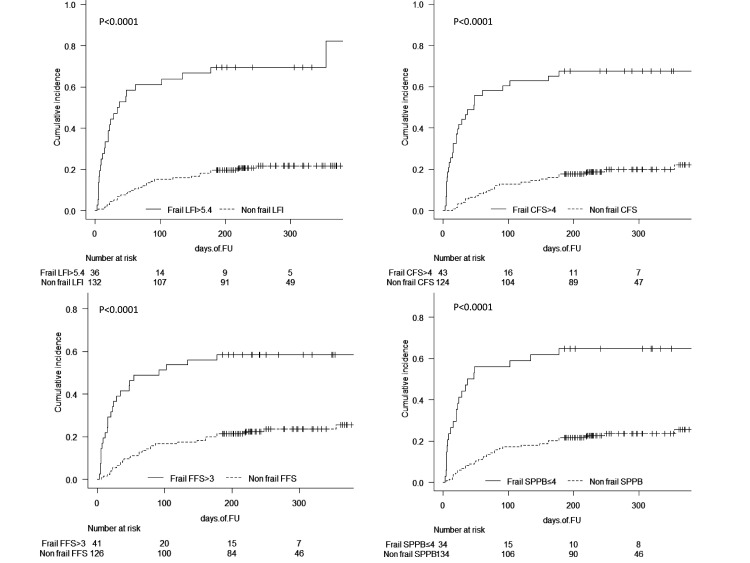
Cumulative incidence of death during follow-up among hospitalized patients with advanced chronic liver disease according to frailty status assessed by four different tools. Upper left pane: Liver Frailty Index (LFI)>5.4; upper right pane: Clinical Frailty Scale (CFS)>4; lower left pane: Fried Frailty Score (FFS)>3; and lower right pane: Short Physical Performance Battery test (SPPB)≤5, P for all curves <0.0001. FU – follow-up.

**Figure 2:**
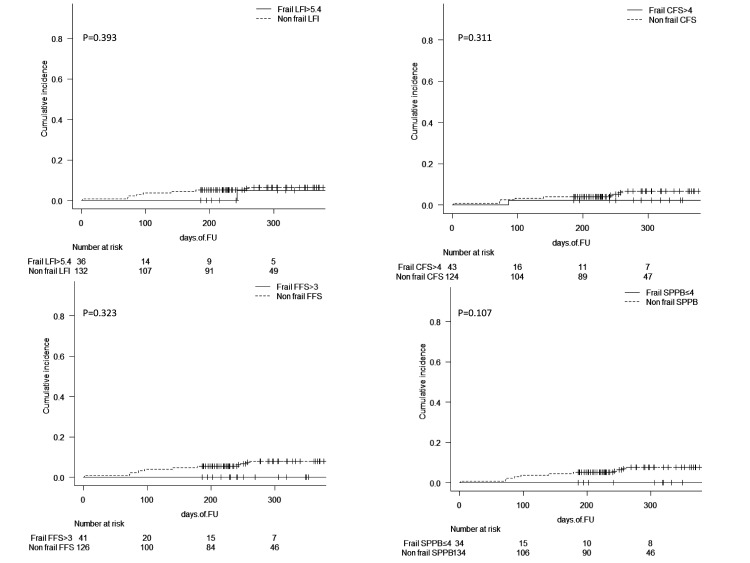
Cumulative incidence of liver transplantation during follow-up among hospitalized patients with advanced chronic liver disease according to frailty status assessed by four different tools. Upper left pane: Liver Frailty Index (LFI)>5.4; upper right pane: Clinical Frailty Scale (CFS)>4; lower left pane: Fried Frailty Score (FFS)>3; and lower right pane: Short Physical Performance Battery test (SPPB)≤4. There was no significant difference for any of the curves. FU – follow-up.

According to the defined score cut-offs, we constructed the cumulative incidence plots for both events (death or LT) for the group of frail patients in comparison with non-frail patients using all four definitions of frailty (Figure 1 and 2).

The predictors of overall survival were assessed with two models. The first was Fine-Gray proportional hazard regression for competing events, in which death was defined as the event of interest and LT as the competing event. The parameters identified as significant predictors of overall mortality in the univariate analysis were entered into a stepwise backward multivariate model corrected for age, which yielded independent predictors of mortality with hazard ratios (HRs) and 95% confidence intervals (CIS). To illustrate the predictive power of frailty among various subgroups (infection at baseline, high MELD score [>20 points], and alcoholic liver disease), we performed a sensitivity analysis yielding HRs and 95% CIs (Supplementary Table 1(Supplementary Table 1)). Second, we used the Cox hazard regression model for the prediction of death or LT in univariate and bivariate analysis (frailty scores and MELD), yielding HRs and 95% CIs for mortality with concordance and its standard deviations (Supplementary Table 2(Supplementary Table 2)). The statistical analysis was carried out with MedCalc, version 13.1.2. (MedCalc Software, Ostend, Belgium) and R v. 3.5.1 using EZR plug-in, version 1.37 (14).

## Results

During the inclusion interval of 13 months, we enrolled 168 patients and followed them for a median of 226.5 (IQR 260) days. At baseline, the median age was 57.9 (IQR 14.3) years, and 109 patients (64.9%) were men. The primary etiology of ACLD was alcohol-related liver disease in 132 patients (78.6%), non-alcoholic steatohepatitis cirrhosis in 20 (11.9%), viral hepatitis B or C related cirrhosis in 9 patients (5.4%), autoimmune syndromes in 8 patients (4.8%), and other etiologies in 11 patients (6.5%). Three patients also had HCC (1.8%). The median MELD score was 16 (IQR 9) (Table 1).

**Table 1:** Baseline characteristics of hospitalized patients with advanced chronic liver disease (ACLD), N = 168*

	Median (interquartile range) or n (%)
**Age, years**	57.9 (14.3)
**Men (%)**	109 (64.9)
**Body mass index, kg/m^2^**	26.0 (5.9)
**Etiology of ACLD**	
**alcohol**	132 (78.6)
**NASH**	20 (11.9)
**viral**	9 (5.4)
**AI**	8 (4.8)
**other**	11 (6.5)
**>1 factor**	12 (7.1)
**Hepatocellular carcinoma (%)**	3 (1.8)
**Serum albumin, g/L**	28 (8)
**Serum sodium, mmol/L**	135 (6)
**C reactive protein, mg/L**	12.5 (19.2)
**Child Pugh Turcotte score**	9.00 (3)
**MELD-Na score**	16.00 (9)
**MELD-Na ≥20 points (%)**	46 (27.4)
**Infection on admission (%)**	54 (32.1)
**Number connection test, s†**	67 (65)
**Hepatic encephalopathy stage grade**	
**0**	121 (72)
**1**	29 (17.3)
**2**	17 (10.1)
**3-4**	1 (0.6)
**Ascites grade**	
**none**	53 (31.5)
**controlled**	54 (32.1)
**refractory**	61 (36.3)
**In hospital days**	6 (8.25)
**Follow-up event and duration, n (%), days**	
**none**	104 (61.9), 300.5
**liver transplantation**	10 (6.0), 117.5
**death**	54 (32.1), 35.5
**Mortality**	
**in hospital**	15 (9)
**30 days**	24 (14.3)
**90 days**	41 (24.4)
**180 days**	51 (30.4)

*Abbreviations: AI – cirrhosis due to autoimmune disease; NASH – cirrhosis due to non-alcoholic steatohepatitis; MELD- Na – Model for End-Stage Liver Disease-Sodium Score.

†N = 157.

During the follow-up, 104 (61.9%) patients survived for more than 180 days, 10 patients (6.0%) underwent LT, and 54 patients (32.1%) died. Fifteen patients died during the hospital stay (9%), 24 died in 30 days (14.3%), 41 in 90 days (24.4%), and 51 in 180 days (30.4%).

Frailty assessment scores and their distributions are shown in Table 2. The results of the AUROC analysis and cut-offs for the prediction of death at various time points for all frailty assessment tools are displayed in Table 3. Considering both analyses, we set the following cut-offs for diagnosing frailty in our cohort: LFI>5.4, CFS>4, FFS>3 (>80th percentile), and SPPB≤4 (<20th percentile).

**Table 2:** Basic statistics for the evaluated frailty scores, N = 168

	Percentiles						
	2.5	5	20	median	80	95	97.5
**Liver Frailty Index**	3.3	3.4	3.9	4.5	**5.4**	6.7	6.8
**Clinical Frailty Scale**	1	1	2	4	**5**	7	7
**Fried Frailty Score**	0	0	1	3	**4**	5	5
**Short Physical Performance Battery**	0	0	**4**	8	11	12	12

**Table 3:** Predictive values of the frailty assessment scores for mortality at various time points, N = 161*

	AUROC†	95% CI	Cut-off	Specificity	Sensitivity	P
**In-hospital mortality**						
**Liver Frailty Index**	0.844	0.778-0.896	>5.1	78.1	86.7	<0.0001
**Clinical Frailty Scale**	0.87	0.808-0.918	>4	80.1	86.7	<0.0001
**Fried Frailty Score**	0.757	0.683-0.822	>3	78.6	66.7	<0.0001
**Short Physical Performance Test**	0.826	0.758-0.881	≤5	80.1	80	<0.0001
						
**Mortality at 30 days**						
**Liver Frailty Index**	0.797	0.726-0.856	>5.7	91.2	66.7	<0.0001
**Clinical Frailty Scale**	0.864	0.801-0.913	>4	83.2	79.2	<0.0001
**Fried Frailty Score**	0.753	0.678-0.819	>3	81.6	66.7	<0.0001
**Short Physical Performance Test**	0.808	0.737-0.866	≤5	82.5	70.8	<0.0001
**Mortality at 90 days**						
**Liver Frailty Index**	0.762	0.688-0.825	>4.5	62.5	80.5	<0.0001
**Clinical Frailty Scale**	0.778	0.706-0.839	>4	83.2	79.2	<0.0001
**Fried frailty score**	0.706	0.629-0.776	>3	82.4	48.8	<0.0001
**Short Physical Performance Test**	0.754	0.679-0.819	≤5	85	56.1	<0.0001
**Mortality at 180 days**						
**Liver Frailty Index**	0.777	0.705-0.839	>4.5	66.4	80.4	<0.0001
**Clinical Frailty Scale**	0.763	0.690-0.827	>4	88.2	56.9	<0.0001
**Fried Frailty Score**	0.711	0.634-0.780	>3	84.4	47.1	<0.0001
**Short Physical Performance Test**	0.747	0.672-0.813	≤5	86.4	51	<0.0001

*Abbreviations: AUROC – area under the ROC curve; CI – confidence interval.

†Excluding patients undergoing liver transplantation within 180 days of follow-up, n = 7.

The cumulative incidence of death or LT according to the presence of frailty is shown in Figures 1 and 2, respectively. When all frailty definitions were used, mortality was significantly higher in frail patients (*P* < 0.0001). The likelihood of LT was lower among frail patients, but the difference was not significant. Since the predictive cut-offs for all the evaluated frailty tools in our sample were higher than the previously reported cut-offs among outpatients, we constructed the cumulative incidence plots for different score cut-offs (Supplementary Figure 1(Supplementary Figure 1)). For LFI (<4.5, 4.5-5.4, >5.4), the three curves had a significantly different cumulative incidence of death. For CFS (0-3, 4, 5-9), FFS (0-2, 3, 3-6), and SPPB (0-4, 5-10, 11-12), the curves were significantly different only between the low-risk and the high-risk groups.

The HRs of frailty scores for the prediction of overall mortality in the competing events analysis are shown in Table 4. In a univariate model, we calculated the HRs for LFI (HR 2.53, CI 1.87-3.42), CFS (HR 1.64, CI 1.38-1.93), FFS (HR 1.68, CI 1.37-2.06), and SPPB score (HR 0.79, CI 0.73-0.85). In the sensitivity analysis, we confirmed the predictive value of all frailty scores across all subgroups, except of FFS and SPPB in non-infected and of FFS in non-alcoholic patients (Supplementary Table 1(Supplementary Table 1)). A multivariate model that included all frailty assessment scores was corrected for the relevant covariates (Table 4) and yielded the following independent predictors of overall mortality: MELD score (HR 1.17, CI 1.12-1.22), LFI (HR 1.41, CI 1.02-1.95), infection on admission (HR 2.32, CI 1.23-4.34), and overt HE (HR 2.39, CI 1.27-4.48).

**Table 4:** Baseline predictors of overall mortality in univariate and multivariate model*

	Univariate			Multivariate		
	HR	95% CI	P value	HR	95% CI	P value
**Age, years^†^**	1.00	0.97-1.02	0.760	1.03	1.0-1.07	0.04
**Female sex**	1.07	0.61-0.9	0.800			
**Body mass index baseline, kg/m^2^**	0.97	0.92-1.02	0.260			
**Etiology alcohol (yes)^†^**	1.23	0.63-2.42	0.550			
**Etiology autoimmune (yes)**	1.80	0.69-4.7	0.230			
**MELD-Na score^†^**	1.17	1.12-1.22	<0.001	1.17	1.12-1.22	<0.001
**Serum albumin^†^**	0.91	0.88-0.94	<0.001			
**Refractory ascites^†^**	1.69	0.99-2.86	0.053			
**Hepatic encephalopathy (overt)^†^**	3.84	2.24-6.58	<0.001	2.39	1.27-4.48	<0.001
**Hepatocellular carcinoma (yes)**	1.01	0.16-6.56	0.990			
**C-reactive protein, mg/L^†^**	1.02	1.01-0.02	<0.001			
**Infection on admission (yes)^†^**	3.94	2.3-6.73	<0.001	2.32	1.23-4.34	0.001
**Liver Frailty Index^†^**	2.53	1.87-3.42	<0.001	1.41	1.02-1.95	0.04
**Clinical Frailty Scale^†^**	1.64	1.38-1.93	<0.001			
**Fried Frailty Score^†^**	1.68	1.37-2.06	<0.001			
**SPPB score^†^**	0.79	0.73-0.85	<0.001			
**Length of hospital stay, days^†^**	1.04	1.02-1.06	<0.001			

*Abbreviations: HR – hazard ratio; CI – confidence interval; MELD-Na – Model for End-Stage Liver Disease-Sodium score; SPPB – Short Physical Performance Battery Score.

**†**Variables in the multivariate model in a competing events analysis for overall survival.

In the Cox model, in order to determine the predictors of death or LT, we compared prognostic values among the frailty assessment tools in univariate and bivariate analysis (frailty tool + MELD). All frailty assessment tools were significant predictors of mortality, with LFI having the highest numerical concordance value alone and also in combination with the MELD score (Supplementary Table 2(Supplementary Table 2)).

## Discussion

Our study showed that frailty could be successfully assessed in all patients using all the tested tools, while frail patients had an overall worse prognosis. A multivariate model using all the tools corrected for age and disease complications pointed to LFI, MELD score, overt HE, and infection at baseline as independent predictors of death. Among the frailty assessment tools, LFI alone and LFI in combination with MELD also had the highest numerical predictive value for death in transplant-free survival analysis. Our study confirms the feasibility of use of frailty among hospitalized patients with ACLD regardless of the used assessment tool, as well as its good prognostic value. Furthermore, LFI had the most convenient combination of applicability and accuracy for the initial and follow-up assessment.

Identifying frailty at hospitalization for cirrhosis decompensation is important since it may offer an opportunity for early interventions. A large body of evidence exists for the prognostic value of frailty among waitlisted outpatients (6,8,15,16). For hospitalized ACLD patients, the prognostic value of frailty for 90-day mortality and the length of stay has also been confirmed (17). The study used “standard” frailty assessment tools for inpatients, such as activities of daily living or the Braden scale (17). The present study tested the diagnostic instruments from the evolving frailty toolkit that had been previously validated for ACLD outpatients: two observational scales (CFS and FFS) and two objective scores (LFI and SPPB). We aimed to assess their validity and suitability for our daily clinical practice (8,10,15,16). The definition of frailty was derived from previous studies in terms of a range of values and scores higher than the 80th or lower than the 20th percentile (8,15). To make our sample as similar as possible to our real-life case-mix, we decided to use as few pre-defined exclusion criteria as possible. We did not apply the exclusion criteria used in previous studies such as HE grade 2 and 3, MELD<12, extra MELD points for HCC, and the same-day rule for the frailty test and physical examination (18). Gait-speed scores were often not measured, but they were entered according to the pre-defined consensus. We hypothesized that the frailty scores validated mostly in waitlisted outpatients would retain their prognostic values in inpatients, and we confirmed the hypothesis regardless of the tool used.

Not surprisingly, LFI values histogram shifted to the right, yet the LFI values fitted to the range of values from previous studies, lending support to the reproducibility of LFI (19). We showed that the LFI cut-off for frailty derived from our percentile distribution curve (>5.4) predicted the prognosis in addition to the previously defined range for frailty (4.5-5.4). This finding demonstrates the predictive power of LFI in its entire range, as well as the absence of its ceiling effect. As for CFS, one study has reported that the cut-off >4 identified 18% of frail transplant candidates and highlighted CFS (with MELD) as a more accurate predictor of hospitalization and death compared with FFS and SPPB (8). In the present study, CFS also had the highest prognostic value for mortality during the hospital stay and at 30 and 90 days. FFS is the first frailty assessment tool translated from geriatrics (with the cut-off for frailty ≥3) (20). We identified a higher FFS cut-off (>3) and the lowest numerical prognostic value among all the tested scores. As for SPPB, we demonstrated a lower cut-off (<5), but a good prediction of survival. These results prove the applicability and prognostic value of the tested frailty instruments in a real-life sample of hospitalized ACLD patients. They were, however, not exclusive enough to allow us to decide which of them would fulfill the specific demands posed by a low-resource clinical setting. Subjective and observational scales have the advantages of rapidity and simplicity, while objective measures are more utilitarian in the complicated decision-making process, such as prioritizing on the waiting list for LT or switching to palliative care. In addition, disease evolution often affects physiological reserves in both directions. These changes could be better captured by instruments allowing a repeated assessment, as well as by objective and sensitive tools (16). LFI was superior to SPPB in most aspects, thus it appears to be the most applicable and accurate tool for repeated frailty assessment among hospitalized patients.

In the sensitivity analysis, we evaluated the risk of death in frail patients subdivided according to alcoholic etiology, MELD score, and infection at baseline, demonstrating that frailty represented a risk factor of death in all subgroups. Of note is our observation that frail patients with MELD≥20 or infection at baseline had a multiplied risk of death. This finding might suggest that the therapeutic care during hospitalization should be focused mainly on the recovery from the decompensating event. Frailty diagnosed in this clinical context could also provide an indispensable argument to either prioritize the patients who are too sick to be transplanted on the waiting list or refer them to palliative care (21,22). Among patients with MELD score <20, frailty increased mortality prediction 2.5- to 5-fold. This observation shows that the prediction based on MELD alone underestimates the risk of death and should be combined with frailty in risk assessment. In a bivariate analysis of transplant-free survival, the combination of the LFI and MELD had the highest numerical predictive power for mortality compared with the other tools.

Finally, the question arises as to the suitable intervention after diagnosing frailty. In contrast to patients who are not candidates for LT, for waitlisted patients there is recall policy after the detection of functional decline. Both groups, however, would benefit from diagnosing frailty and its progression early in the chain of care. There is evidence on the efficacy of interventions that would prevent further functional decline or would improve frailty (23,24). A beneficial impact of exercise rehabilitation programs (25) and/or nutritional support, such as the use of branched-chain amino-acid supplements (26), has been recently reported. Multi-disciplinary management of the ongoing complications, as well as nutritional support according to the recently upgraded guidelines of the European Society for the Study of the Liver, should be recommended (27). In addition, referring cirrhotic patients to palliative care improves the quality of life and decreases the cost of care, with no increase in mortality or the likelihood of LT (28,29).

Our study has some limitations. It is a single-center study conducted at a tertiary referral liver unit with a low-volume LT center, which makes it subject to selection bias. Therefore, our conclusions merit further validation in a multicenter setting. The present study also does not report on sarcopenia, not allowing for a comparison between prognostic values of sarcopenia and frailty. Nevertheless, the recently updated report of the European Working Group on Sarcopenia in Older People has given the priority to muscle strength in the new definition of sarcopenia, thus bringing it closer to the definition of frailty. Finally, for logistic reasons, we did not evaluate frailty by all the available assessment tools, such as activities of daily living or Karnofsky index. In contrast with US hospitals, Karnofsky index is not standardly recorded at hospital admission in Slovakian hospitals (30,31).

In conclusion, this study demonstrates the feasibility of using LFI, CFS, FFS, or SPPBT to diagnose frailty among inpatients with cirrhosis in clinical practice. Frailty provides important prognostic information and was proven to be an independent predictor of mortality. Among the used tools, LFI and CFS yielded the highest numerical predictive value. Our study lends support to the standardized assessment of frailty in all hospitalized cirrhotic patients and to the consensus on the universal diagnostic instrument.
