# Supplementary material for: Applicability and prognostic value of frailty assessment tools among hospitalized patients with advanced chronic liver disease
Source: Croat Med J. 2021 Feb;62(1):8–16. doi: 10.3325/cmj.2021.62.8 (PMC7976891; doi:10.3325/cmj.2021.62.8)
Supplement: Supplementary Table 1 [file CroatMedJ_62_s001.pdf]

Figure 1.

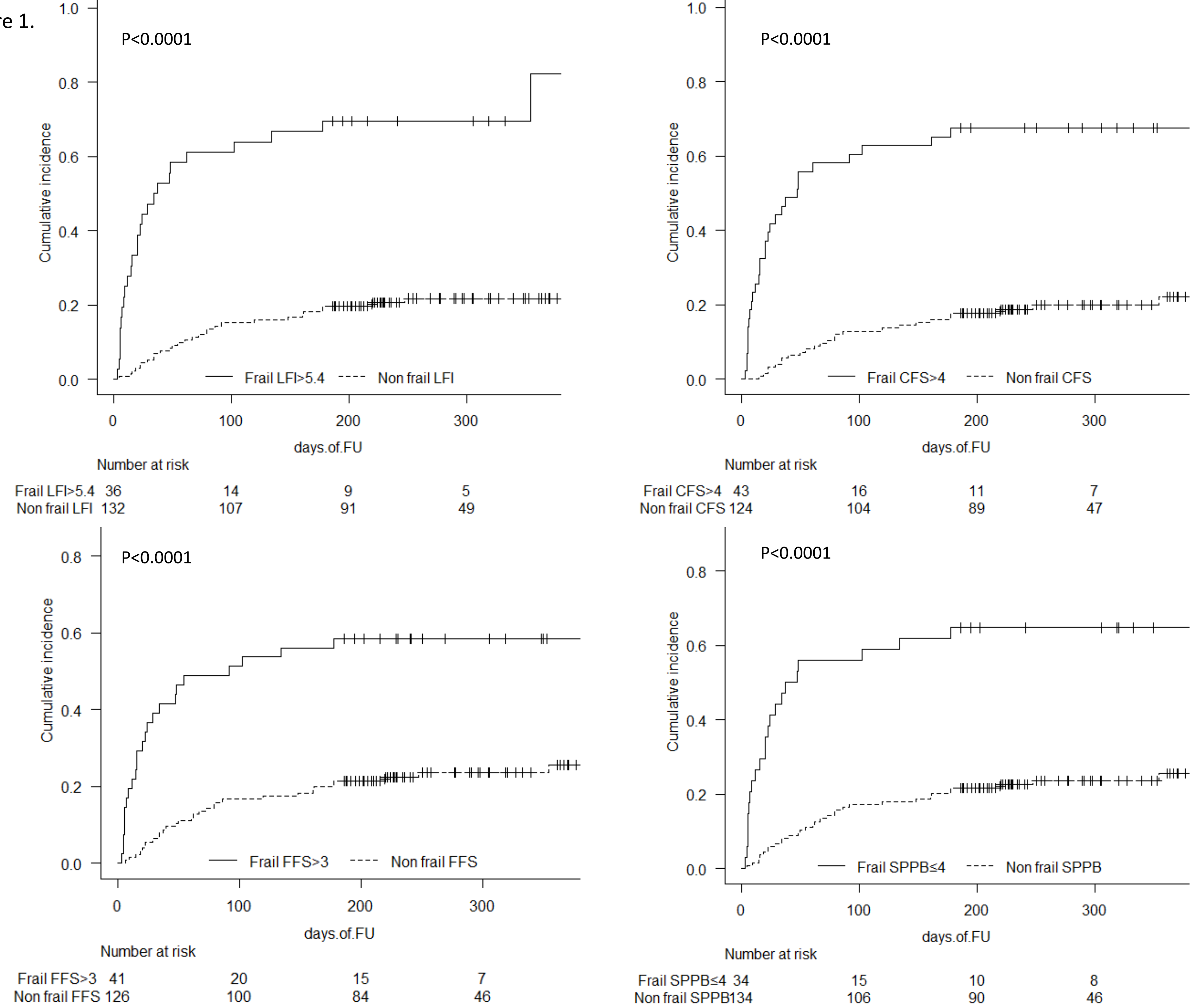

P<0.0001
Cumulative incidence
days.of.FU
Frail LFI>5.4
Non frail LFI
Number at risk
Frail LFI>5.4 36 14 9 5
Non frail LFI 132 107 91 49
P<0.0001
Cumulative incidence
days.of.FU
Frail CFS>4
Non frail CFS
Number at risk
Frail CFS>4 43 16 11 7
Non frail CFS 124 104 89 47
P<0.0001
Cumulative incidence
days.of.FU
Frail FFS>3
Non frail FFS
Number at risk
Frail FFS>3 41 20 15 7
Non frail FFS 126 100 84 46
P<0.0001
Cumulative incidence
days.of.FU
Frail SPPB≤4
Non frail SPPB
Number at risk
Frail SPPB≤4 34 15 10 8
Non frail SPPB134 106 90 46

Figure 2.

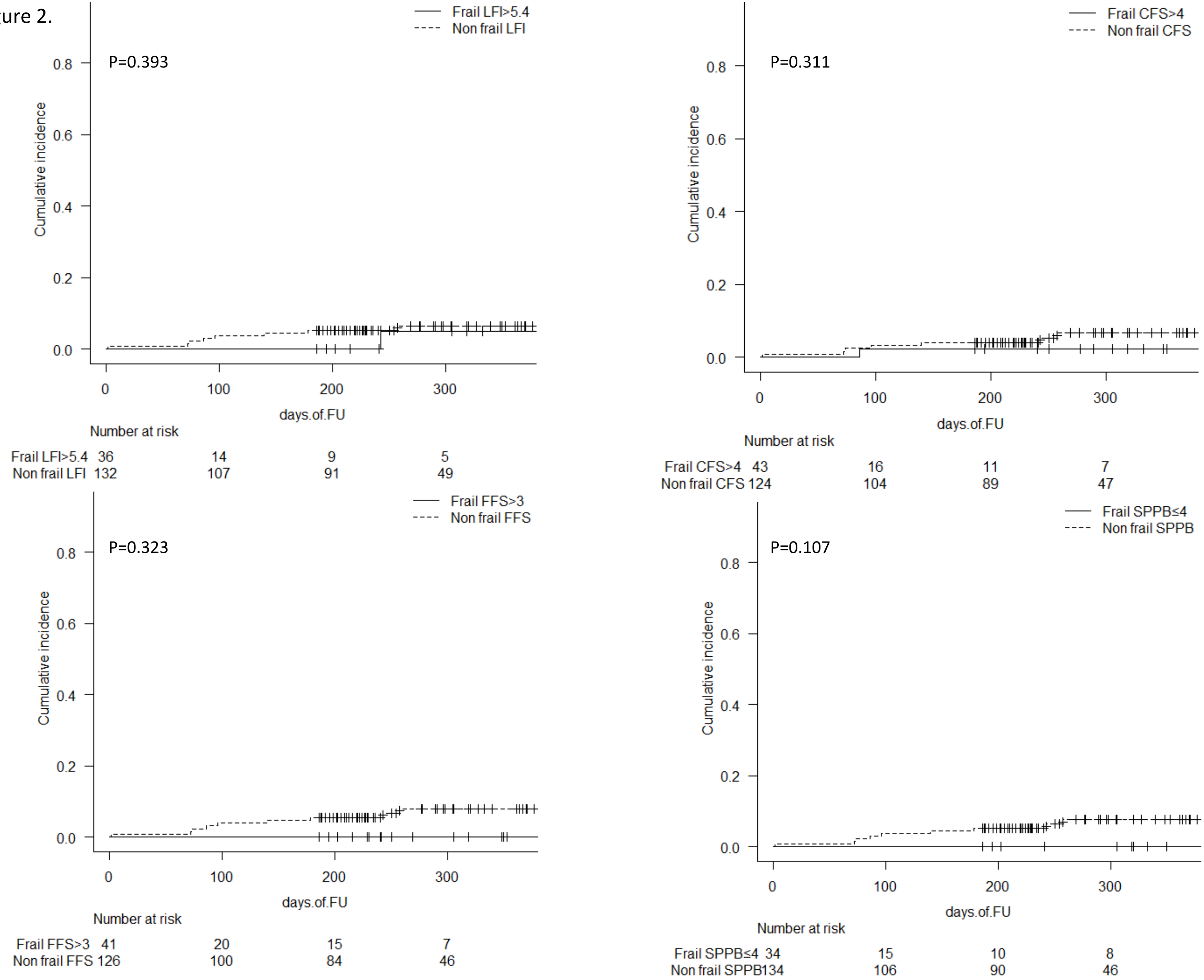

Frail LFI>5.4
Non frail LFI
P=0.393
Cumulative incidence
0.0
0.2
0.4
0.6
0.8
0
100
200
300
days.of.FU
Number at risk
Frail LFI>5.4 36 14 9 5
Non frail LFI 132 107 91 49
Frail CFS>4
Non frail CFS
P=0.311
Cumulative incidence
0.0
0.2
0.4
0.6
0.8
0
100
200
300
days.of.FU
Number at risk
Frail CFS>4 43 16 11 7
Non frail CFS 124 104 89 47
Frail FFS>3
Non frail FFS
P=0.323
Cumulative incidence
0.0
0.2
0.4
0.6
0.8
0
100
200
300
days.of.FU
Number at risk
Frail FFS>3 41 20 15 7
Non frail FFS 126 100 84 46
Frail SPPB≤4
Non frail SPPB
P=0.107
Cumulative incidence
0.0
0.2
0.4
0.6
0.8
0
100
200
300
days.of.FU
Number at risk
Frail SPPB≤4 34 15 10 8
Non frail SPPB134 106 90 46

Figure S1.

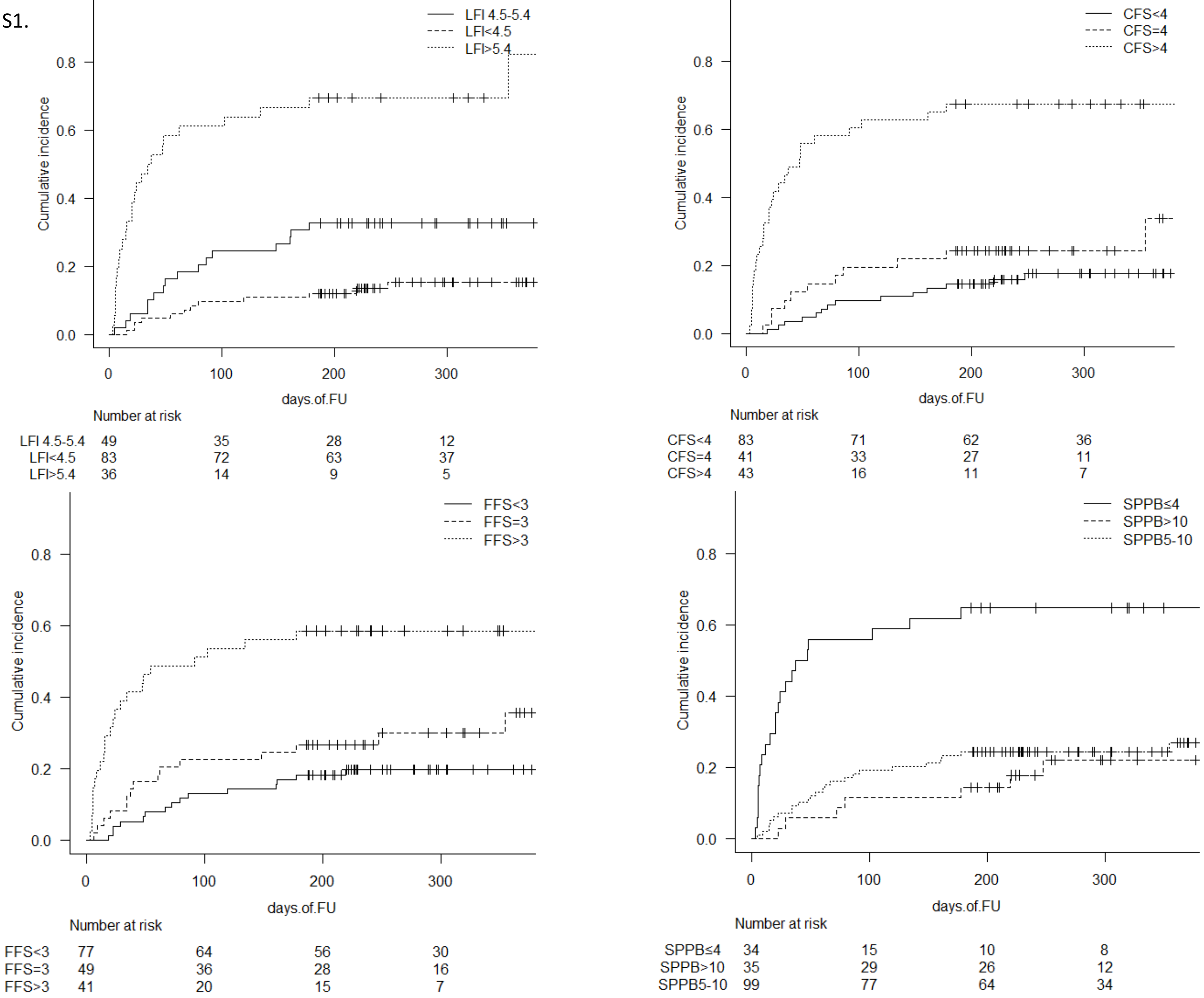
LFI 4.5-5.4
LFI<4.5
LFI>5.4
0.8
0.6
0.4
0.2
0.0
Cumulative incidence
0
100
200
300
days.of.FU
Number at risk
LFI 4.5-5.4 49 35 28 12
LFI<4.5 83 72 63 37
LFI>5.4 36 14 9 5
CFS<4
CFS=4
CFS>4
0.8
0.6
0.4
0.2
0.0
Cumulative incidence
0
100
200
300
days.of.FU
Number at risk
CFS<4 83 71 62 36
CFS=4 41 33 27 11
CFS>4 43 16 11 7
FFS<3
FFS=3
FFS>3
0.8
0.6
0.4
0.2
0.0
Cumulative incidence
0
100
200
300
days.of.FU
Number at risk
FFS<3 77 64 56 30
FFS=3 49 36 28 16
FFS>3 41 20 15 7
SPPB≤4
SPPB>10
SPPB5-10
0.8
0.6
0.4
0.2
0.0
Cumulative incidence
0
100
200
300
days.of.FU
Number at risk
SPPB≤4 34 15 10 8
SPPB>10 35 29 26 12
SPPB5-10 99 77 64 34
