# Supplementary material for: Applicability and prognostic value of frailty assessment tools among hospitalized patients with advanced chronic liver disease
Source: Croat Med J. 2021 Feb;62(1):8–16. doi: 10.3325/cmj.2021.62.8 (PMC7976891; doi:10.3325/cmj.2021.62.8)
Supplement: Supplementary Table 2 [file CroatMedJ_62_s002.pdf]

Supplementary Table 1

Table S1.
Frailty as a predictor of mortality in selected patient subgroups, a sensitivity analysis*

	HR	95%CI	P value
Subgroup		*Frailty assessment tool & cut-off*	
		Liver frailty index >5.4	
not infected	3,7	1.52-9.34	0,005
infected	4,96	2.46-10.28	<0.001
non-ALD	16,8	4.49-65.12	<0.001
ALD†	4,89	2.72-8.97	<0.001
MELD<=20	5,03	2.29-11.31	<0.001
MELD>20	5,18	2.32-11.62	<0.001
		Clinical frailty scale >4	
not infected	4,45	1.76-11.52	0,002
infected	3,84	1.85-8.02	<0.001
non-ALD	7,03	2.03-24.37	0,002
ALD	5,5	3.07-10.09	<0.001
MELD<=20	3	1.21-7.34	0,016
MELD>20	5,56	2.63-12.16	<0.001
		Fried frailty score >3	
not infected	1,75	0.61-4.97	0,3
infected	4	2.05-7.97	<0.001
non-ALD	1,99	0.64-6.87	0,28
ALD	4,5	2.54-8.18	<0.001
MELD<=20	2,71	1.19-6.26	0,021
MELD>20	4,08	1.88-8.94	<0.001
		Short performance physical battery test≤4	
not infected	1,83	0.57-5.69	0,3
infected	4,62	2.28-9.32	<0.001
non-ALD	4,94	1.42-17.38	0,013
ALD	4,25	2.91-7.87	<0.001
MELD<=20	2,57	0.97-6.62	0,051
MELD>20	3,35	1.64-7.12	0,002

**Competing events analysis for death during follow-up*

† ALD: alcoholic liver disease
