# Supplementary material for: Applicability and prognostic value of frailty assessment tools among hospitalized patients with advanced chronic liver disease
Source: Croat Med J. 2021 Feb;62(1):8–16. doi: 10.3325/cmj.2021.62.8 (PMC7976891; doi:10.3325/cmj.2021.62.8)
Supplement: Supplementary Figure 1 2 [file CroatMedJ_62_s003.pdf]

Supplementary Table 2.

Table S2.
Univariate and bivariate transplantation-free survival analysis* among 168 patients hospitalized for ACLD†

	HR	95% CI	P value	Concordance
Univariate				
Age	0,99	0.97-1.01	0,37	0.519±0.4
C reactive protein	1,01	1.01-1.02	<0.001	0.687±0.04
Liver frailty index	2,22	1.72-2.86	<0.001	0.712±0.04
Clinical frailty scale	1,52	1.31-1.75	<0.001	0.701±0.04
Fried frailty score	1,53	1.25-1.87	<0.001	0.663±0.04
Short physical perf. test	0,82	0.77-0.88	<0.001	0.681±0.04
MELD score	1,14	1.1-1.18	<0.001	0.723±0.04
Child-Pugh-Turcotte score	1,52	1.34-1.72	<0.001	0.759±0.04
Bivariate				
Liver frailty index	1,87	1.44-2.44	<0.001	0.771±0.04
MELD score	1,12	1.1-1.16	<0.001	
Clinical frailty scale	1,32	1.13-1.54	<0.001	0.75±0.04
MELD score	1,11	1.07-1.16	<0.001	
Fried frailty score	1,48	1.19-1.83	<0.001	0.76±0.04
MELD score	1,13	1.09-1.17	<0.001	
Short physical perf. test	0,87	0.81-0.93	<0.001	0.75±0.04
MELD score	1,12	1.07-1.16	<0.001	

* *Cox proportional hazard regression for the risk of death or liver transplantation*

† ACLD: advanced chronic liver disease
